# Users’ perception of the OH-EpiCap evaluation tool based on its application to nine national antimicrobial resistance surveillance systems

**DOI:** 10.3389/fpubh.2023.1138645

**Published:** 2023-06-19

**Authors:** Pedro Moura, Lucie Collineau, Marianne Sandberg, Laura Tomassone, Daniele De Meneghi, Madelaine Norström, Houda Bennani, Barbara Häsler, Mélanie Colomb-Cotinat, Clémence Bourély, Maria-Eleni Filippitzi, Sarah Mediouni, Elena Boriani, Muhammad Asaduzzaman, Manuela Caniça, Cécile Aenishaenslin, Lis Alban

**Affiliations:** ^1^National Food Institute, Technical University of Denmark, Lyngby, Denmark; ^2^University of Lyon - French Agency for Food, Environmental and Occupational Health and Safety (ANSES), Epidemiology and Surveillance Support Unit, Lyon, France; ^3^Department of Veterinary Sciences, University of Turin, AgroVet campus, Grugliasco-Turin, Italy,; ^4^Norwegian Veterinary Institute, Ås, Norway; ^5^Veterinary Epidemiology Economics and Public Health Group, Department of Pathobiology and Population Sciences, Royal Veterinary College, London, United Kingdom; ^6^Santé publique Franced, Direction des maladies infectieuses, Saint Maurice, France; ^7^French Ministry of Agriculture and Food, General Directorate for Food, Animal Health Unit, Paris, France; ^8^Laboratory of Animal Health Economics, Aristotle University of Thessaloniki, Thessaloniki, Greece,; ^9^Veterinary Epidemiology Unit, Sciensano, Brussels, Belgium; ^10^Faculty of Veterinary Medicine, Université de Montréal, Saint-Hyacinthe, QC, Canada; ^11^EB Consult, Hellebæk, Denmark; ^12^Department of Community Medicine and Global Health, Institute of Health and Society, Faculty of Medicine, University of Oslo, Oslo, Norway; ^13^National Reference Laboratory of Antibiotic Resistances and Healthcare Associated Infections, Department of Infectious Diseases, National Institute of Health Dr. Ricardo Jorge, Lisbon, Portugal; ^14^Department of Food Safety, Veterinary Issues and Risk Analysis, Danish Agriculture and Food Council, Copenhagen, Denmark; ^15^Department of Veterinary and Animal Sciences, University of Copenhagen, Frederiksberg, Denmark

**Keywords:** One Health, antimicrobial resistance, antimicrobial consumption monitoring, system evaluation, surveillance system

## Abstract

**Introduction:**

Antimicrobial resistance (AMR) is a One Health (OH) challenge. To achieve or maintain an effective and efficient AMR surveillance system, it is crucial to evaluate its performance in meeting the proposed objectives, while complying with resource restrictions. The OH-EpiCap tool was created to evaluate the degree of compliance of hazard surveillance activities with essential OH concepts across the following dimensions: organization, operational activities, and impact of the surveillance system. We present feedback on the application of the OH-EpiCap tool from a user’s perspective, based on the use of the tool to evaluate nine national AMR surveillance systems, each with different contexts and objectives.

**Methods:**

The OH-EpiCap was assessed using the updated CoEvalAMR methodology. This methodology allows the evaluation of the content themes and functional aspects of the tool and captures the user’s subjective experiences via a strengths, weaknesses, opportunities, and threats (SWOT) approach.

**Results and Discussion:**

The results of the evaluation of the OH-EpiCap are presented and discussed. The OH-EpiCap is an easy-to-use tool, which can facilitate a fast macro-overview of the application of the OH concept to AMR surveillance. When used by specialists in the matter, an evaluation using OH-EpiCap can serve as a basis for the discussion of possible adaptations of AMR surveillance activities or targeting areas that may be further investigated using other evaluation tools.

## 1. Introduction

International organizations are calling for a One Health (OH) approach to tackle antimicrobial resistance (AMR). The One Health High Level Expert Panel (OHHLEP) defines OH as “an integrated, unifying approach that aims to sustainably balance and optimize the health of people, animals, and ecosystems” recognizing that the health of these populations is closely linked and interdependent (1).

AMR genes and microbes know no border, and certain antimicrobial agents are cross used in humans, animals, and plants. Hence, AMR is one of the quintessential examples of a OH challenge (2). Therefore, an integrated, multisectoral approach is necessary to address the issue (3, 4). Integrated surveillance, according to Aenishaenslin et al., is the “systematic collection, analysis, interpretation of data, and dissemination of information collected from different components of a system to provide a global, multidisciplinary, multi-perspective understanding of a health problem and to inform system-based decisions” (5). These actions should be coordinated between the human, animal and environmental sectors (6).

The application of this concept to national surveillance systems is essential to better understand AMR emergence and dissemination and to sustain risk mitigation decisions (7). The OHHLEP has recently released a theory of change for OH that can help to support intersectoral collaboration in national strategies for OH challenges, including those aiming to keep antimicrobials (AM) effective for future generations of people and animals. This theory of change includes the goals, objectives, desired impact at country level, intermediate outcomes, and related functions (1).

Conducting regular evaluations of a surveillance system’s processes and performance is crucial to assess if the established objectives are being met in the most cost-effective way (8). OH initiatives should preferably be evaluated using a methodology that targets all disciplines encompassed and estimate the potential added value of the current approach over a less integrated one (9). The objectives of the evaluation should be made clear from the start, and an overview of the systems’ surveillance components should be produced to guide it, and to balance the objectives of the evaluation with the available resources to perform it (8).

The international network CoEvalAMR was established in 2019 with the goal of providing guidance to help users in choosing an assessment tool from a catalog of tools available to evaluate antimicrobial use (AMU) and AMR surveillance systems (10). Moreover, the network aimed to guide future applications and improvement of the tools assessed and the development of new tools. To meet these aims, a methodology focusing on the users’ perception of the tool was developed during Phase 1 of the CoEvalAMR network (11). The original methodology was used by Sandberg et al. (11) to provide feedback on six different evaluation tools based on their application in eight countries. Based on the experience gained, the methodology has recently been updated and further refined, as part of the work undertaken in Phase 2 of the CoEvalAMR network (12). The methodology encompasses the evaluation of descriptive and functional aspects, together with an assessment of content themes and questions on strengths, weaknesses, opportunities, and threats (SWOT) (12).

OH-EpiCap is among the catalog of tools being assessed in Phase 2 of the CoEvalAMR network. This tool has recently been developed by the MATRIX consortium, funded by the One Health European Joint Program to systematize the characterization of epidemiological surveillance activities in a national surveillance system (13). OH-EpiCap is presented as an easy-to-apply tool, covering previously overlooked aspects such as the impact of integrated surveillance. More specifically, the purpose of OH-EpiCap is to facilitate the evaluation and reinforcement of national capacities and capabilities for OH integrated surveillance of zoonotic hazards (13).

In this study, we applied and evaluated OH-EpiCap using the updated CoEvalAMR user’s perception methodology and presented feedback on the application of the OH-EpiCap tool to nine national AMR surveillance systems, with different monitoring contexts and objectives.

## 2. Materials and methods

### 2.1. Description of OH-EpiCap

The OH-EpiCap tool is composed of three thematic domains (called “dimensions”), each with four different targets that are again segmented into four indicator questions, leading to a total of 48 indicators, briefly presented in Table 1. Each indicator is scored from 1 (no compliance) to 4 (full compliance), with the possibility to select “non-applicable” in case the indicator is not relevant to the system under evaluation. All indicators have the same weight, and for each target, the average value of the indicators’ scores is converted into a target score (13).

**Table 1:** Dimensions, targets and indicators evaluated by the OH-EpiCap tool—modified after (14).

**Dimension 1: Organization**			
**Target 1.1 Formalization:** common aim, support documentations, shared leadership, and definition of roles/composition of coordination committees	**Target 1.2 Coverage:** inclusion of all relevant actors, disciplines, sectors, geography, populations, and related hazards	**Target 1.3 Resources:** budget and human resources, program training, and sharing of resources	**Target 1.4 Evaluation and resilience:** internal and external evaluations, development/ implementation of corrective measures, and adaptability to change

**Dimension 2: Operational activities**			
**Target 2.1 Data collection and methods sharing:** multisectoral collaboration in the design of surveillance protocols and data collection, harmonization of laboratory techniques and data warehousing	**Target 2.2 Data sharing:** data sharing agreements, assessment of data quality, usefulness of shared data, and the compliance of data with the FAIR (findability, accessibility, Interoperability and Reusability) principle	**Target 2.3 Data analysis and interpretation:** multisectoral integration for data analysis, sharing of analysis techniques, sharing of scientific expertise, and harmonization of indicators	**Target 2.4 Communication:** internal and external communication, dissemination to decision-makers, and information sharing in case of suspicion/particular events

**Dimension 3: Impact**			
**Target 3.1 Technical outputs:** timely detection of emergence, epidemiological knowledge improvement, increased effectiveness of surveillance, and reduction of operational costs	**Target 3.2 Collaborative added value:** strengthening of the OH team and network, international collaboration, and common strategy (road map) design	**Target 3.3 Immediate and intermediate outcomes:** advocacy, awareness, preparedness, and interventions based on the information generated	**Target 3.4 Ultimate outcomes:** research opportunities, policy changes and behavioral changes and better health outcomes

Different respondents can have diverging opinions on the scoring of the indicators that compose OH-EpiCap, according to their backgrounds, perceptions, and expectations. To reduce the possible bias that the subjectivity of the scoring method may create, a consensus among respondents within one working group is required to select a final score among those described in the scoring guide (13).

The tool also includes a graphical interface developed in RShiny, where the results of the evaluation are presented in a dashboard that can be exported as a report. The OH-EpiCap tool is available on the following website: https://freddietafreeth.shinyapps.io/OH-EpiCap/.

### 2.2. Data collection

The nine surveillance systems evaluated were selected by members of the CoEvalAMR network. The selection was made by convenience of the members, due to direct acquaintance with the systems evaluated or close personal contacts. The evaluations were conducted from August to November 2022.

The number of respondents involved in the evaluation of each case study varied from one to five; these respondents are referred to as “assessors” throughout the text. The assessors filled in the OH-EpiCap evaluation questions during either a single or repeated workshop session that lasted a total of 2–8 h. All assessors involved had expertise in AMR surveillance in the country they represented for this study, scoring the indicator questions according to their own experience or knowledge from previous activities. This methodology makes the evaluation outputs somewhat subjective. In the country case studies that were conducted by more than one assessor, the subjectivity was reduced because of the requirement to reach consensus within the group of assessors who formed part of the country case. Whenever needed, additional experts and information sources were consulted.

The OH-EpiCap tool was used to evaluate national AMR surveillance systems in Bangladesh, Belgium, Canada, Denmark, France, Italy, Norway, Portugal and the United Kingdom (Table 2). The number of assessors and their affiliation, the type of workshop conducted, and the total duration of the evaluation are described for each country in Supplementary Table S1. The surveillance system evaluated in each country including its main aims can be found in Table 2.

**Table 2:** National AMR surveillance systems evaluated using the OH-EpiCap tool.

Country	Name of the system	Main aims of the system
Bangladesh	One Health Event Based Surveillance (EBS)	Develop a ‘One Health surveillance system platform’ to enable early detection of disease outbreaks. Coordinated joint response to disease outbreaks
Belgium	AMR-AMU surveillance program in the context of developing the OH AMU-AMR national report (OH belmap)	Summarize results and trends of existing monitoring programs: related to the consumption of antibiotic agents for food animals and humans and to the monitoring occurrence of antimicrobial resistance in bacteria isolated from food animals, humans and food of animal origin Identify blind spots in monitoring programs and make recommendations to improve future monitoring
Canada	Canadian Integrated Program for Antimicrobial Resistance Surveillance (CIPARS)	Provide an integrated approach to monitor trends in antimicrobial resistance and antimicrobial use in humans & animals and help identify appropriate measures to contain the emergence and spread of resistant bacteria between animals, food, and people in Canada Facilitate assessment of the public health impact of antimicrobials used in humans & agriculture to support the creation of evidence-based policies to control AMU in hospital, community, and agricultural settings Provide timely analysis and dissemination of surveillance data to stakeholders, and facilitate knowledge translation via targeted communications products Allow accurate comparisons with other countries that use similar surveillance systems (NARMS, DANMAP) Provision of data for Health Canada—Veterinary Drugs Directorate for new antimicrobial drug approval processes and post-approval monitoring
Denmark	Danish Program for surveillance of antimicrobial consumption and resistance in bacteria from food animals, food and humans (DANMAP)	Monitor the consumption of antimicrobial agents for food animals and humans and the occurrence of antimicrobial resistance in bacteria isolated from food animals, food of animal origin and humans Study associations between antimicrobial consumption and antimicrobial resistance Identify routes of transmission and areas for further research studies
France	Surveillance system for AMR, AMU and antimicrobial residues	Monitor trends of AMU and AMR in humans and animals, incl. in diseased animals Assess what is common to several sectors and what is not Inform policy recommendations and assess the impact of interventions
Italy	ClassyFarm	Risk categorization of farms according to an integrated approach containing biosecurity, welfare, AMU/AMR, animal health and lesions at slaughterhouse
Norway	The surveillance program for antimicrobial resistance in human pathogens (NORM) and the monitoring program for antimicrobial resistance in bacteria from feed, food and animals (NORM-VET)	NORM: Collect and process data about antibiotic resistance of microbe isolates to determine the incidence and prevalence of antibiotic resistance and monitor changes over time Drive, promote and provide a basis for research to understand why microbes develop antibiotic resistance, with a view to promoting and developing preventive measures in the treatment of infectious diseases Provide a basis to give health advice and information on measures that could prevent development antimicrobial drug resistance to the public and local, regional and central health authorities Give the Norwegian health authorities a foundation to contribute to international statistics within specific areas NORM-VET: Provide and present data on the occurrence and distribution of antimicrobial resistance over time. Describe the relationship between the use of antimicrobials and occurrence of resistance in the veterinary and food production sectors. The information generated is used for research, setting policies, assessing risks, and evaluating interventions
Portugal	Infection Prevention and Control and Antimicrobial Resistance Program (PPCIRA)	Monitor the occurrence of antimicrobial resistance in bacteria isolated from humans Identify routes of transmission Detect and monitor outbreaks caused by bacteria with antimicrobial resistant genes Prevent the emergence and transmission of bacteria with antimicrobial resistant genes
United Kingdom	Surveillance system for AMU and AMR in the UK	Monitor AMU in humans and animals Monitor trends of AMR in bacteria isolated from humans, food producing animals, and food of animal origin Detect new and emerging AMR threats Inform policy recommendations and assess the impact of interventions

### 2.3. Data analysis

The updated CoEvalAMR users’ perception methodology was used to evaluate the OH-EpiCap tool (13). The methodology consists of a series of questions related to: (1) a general description of the case study and the tool, (2) two standardized scoring schemes, one regarding functional aspects, and another for content themes, as well as (3) a SWOT analysis (12). The functional aspects encompassed in the methodology are grouped into: Ease of use, Scope, Prerequisites before use, Time and resources, and Outputs. The content themes related to the tool’s scope are: AMU and AMR, Collaboration, Resources, Output and use of information, Integration, Adaptivity, Technical operations, Impact and Governance. The definitions of the content themes and functional aspects can be consulted in Alban et al. (12).

**Figure 1:**
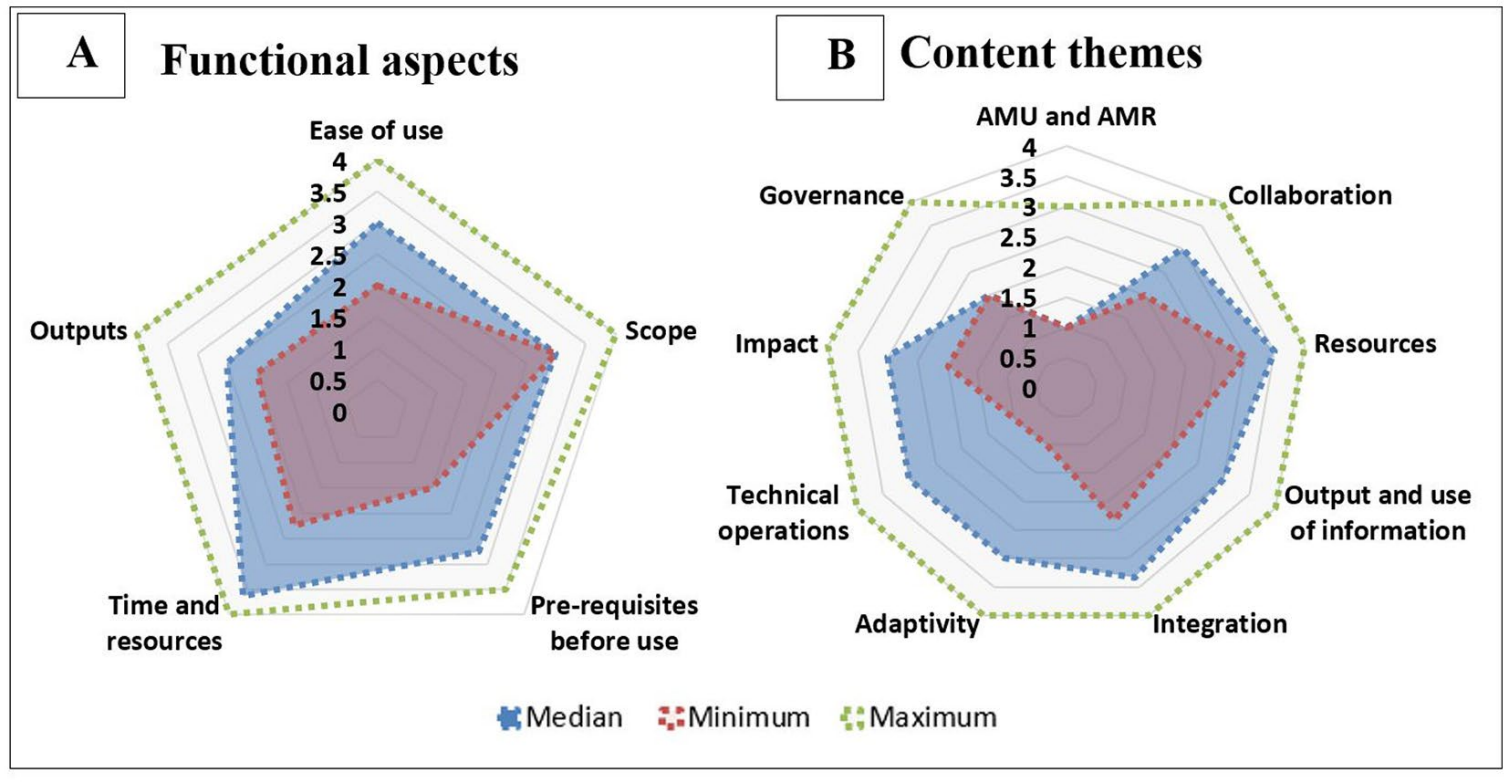
Evaluation of the functional aspects **(A)** and content themes **(B)** of the OH-EpiCap tool according to the CoEvalAMR user’s perception methodology based upon nine country case studies.

Both functional aspects and content themes of OH-EpiCap tool were scored semi-quantitatively using a scale from 1 to 4 or “non-applicable.” Groups composed of several functional aspects or content themes were averaged. Next, the median, maximum and minimum of the scores given by the assessors for functional aspects and content themes were displayed in a radar diagram in Figures 1A,B, respectively. Due to the skewness of the distribution of the answers’ scores, which were not normally distributed, the decision was made to show the median of the scores. Microsoft Excel^®^ was used for data analysis and visualization of the outputs.

The SWOT analysis was undertaken to capture the assessors’ subjective experiences when applying OH-EpiCap. More specifically, the following wording accompanied each component: Strengths: “The strengths of this tool are,” Weaknesses: “The weaknesses of this tool are,” Opportunities: “The added value(s) of using this tool is” and Threats: “This tool might be criticized because of.” A qualitative analysis of the feedback provided by the assessors was performed following the same principles as described by Sandberg et al. (11), which were based on grounded theory (15): all individual sentences were collected, then, sentences with similar content were simplified and condensed into one sentence. The synthesis was performed by three of the assessors and later verified by the remaining assessors.

## 3. Results

### 3.1. Functional aspects

Regarding ease-of-use, OH-EpiCap scored highly due to its user-friendly interface with checkboxes to answer the questions that formed these indicators. The scope of the tool is defined as the ability to address the stated evaluation objectives and is further divided into the content themes evaluated (12). OH-EpiCap was not created with the objective of covering all the national capacities and capabilities for OH integrated surveillance. OH-EpiCap does not cover certain content themes in the level of detail perceived as relevant by the assessors, even for a macro evaluation, as addressed in section 3.2.

The OH-EpiCap tool is free of use. As for prerequisites to use it, no previous data collection is required, and the answers can be given based on the evaluators’ experience connected with the surveillance system. Most indicators require that the evaluation is conducted with specialists in the surveillance system, or that they are consulted in the process, given that an in-depth perspective of the whole surveillance system is needed. No training is necessary to get acquainted with the tool.

OH-EpiCap can—in most cases—be successfully applied by a small group composed of for example three or four persons, providing that the group can form a clear cross-sectoral picture of the surveillance system. Based on our experience, and depending on the expertise of the stakeholders gathered, the evaluation can be conducted in half a day or slightly longer. If additional stakeholders need to be consulted after the initial workshop, the evaluation process will be prolonged. In the case that supplementary information that may impact a given answer is gathered via extra communications, outside of the stakeholder workshop, it should be further discussed with all the assessors.

The graphical outputs generated by the tool were found to provide an easily accessible overview of the responses given. However, given the superficiality of the evaluation content (Table 1), the output of the evaluation need to be further discussed and investigated with relevant actors before it can be translated into specific changes in the surveillance system. Please see the section 3.2 for an elaboration of this issue.

### 3.2. Content themes

The tool does not encompass indicators specifically addressing AMU and AMR surveillance. Even though not covered to a complete extent, OH-EpiCap still provides an overview of the thematic areas connected with the human and budget resources needed to maintain the surveillance activity, as well as the collaboration in the governance structures of the system and in the technical surveillance activities. It also encompasses indicators related to the possible adaptation of the surveillance activities to new challenges and in an efficient manner. The overall impact of the surveillance system is also covered, but without details on how the information generated by the surveillance system could lead to changes in the health outputs. Moreover, the tool does not go into details in the governance domain, specifically the accountability of stakeholders, the coordination of activities and the transparency of processes are only superficially covered.

### 3.3. SWOT analysis

The subjective experience of the application of OH-EpiCap by the assessors captured via SWOT analysis is presented in Table 3 in a summarized format.

**Table 3:** Outcome of SWOT analysis of the OH-EpiCap tool, based on an application of the tool to nine country cases.

Topic and meaning	Synthesis of the comments provided
Strengths: The strengths of this tool are	A feasible compromise between comprehensiveness in quantity of information captured and human/time resources required to carry out evaluation. Simple and well-organized design, following a user-friendly step-by-step approach with boxes to check. No previous extensive training is needed to use it. The provided glossary encompassing explanations of what is meant by an expression is very helpful and increases the ease and swiftness of use. Produces visually attractive figures, encompassed in a report, which provide a good overview of the answers given and make it easy to share and communicate the results. An example of which can be seen in Supplementary Figure S1. In the report, general suggestions for further improvements and indicators of good adherence to OH principles are provided. It is available for free, useful for single or multidisciplinary settings and suitable for any country. It could produce a lot of food for thought, if people with a deep understanding of the surveillance system and all the main processes are consulted.
Weaknesses: The weaknesses of this tool are	Some of the indicator questions could be further simplified to facilitate their interpretation. Although comprehensive, the evaluation products are superficial, and they cannot be directly translated into action, requiring further investigation. If surveillance initiatives are based on one dominating OH pillar, it is not easy to answer some indicator questions, which are structured to catch multi-sectoral/disciplinary collaborations. Some indicators are difficult to score without dedicated *ad-hoc* studies. Sometimes difficult to delineate which impacts comes from OH surveillance versus sectoral surveillance (Dimension 3). Some indicators aiming at evaluating effectiveness refer more to technical performance of surveillance (sensitivity, timeliness) than its capacity to inform decision-making. The tool is sometimes hard to apply to a system which integrates data from multiple domains such as AMR and AMU in animals and humans, but is managed by only one institution, as several items refer to inter-institutions collaboration and governance.
Opportunities: The added value(s) of using this tool is	Helpful to identify new areas that should be further investigated and to initiate discussion around the possibility of adapting the existing systems. Provides a good overview of a surveillance system targeting one hazard, or a component of a complex system. Evaluation can be performed in a short time, so it may be done frequently, and after relevant updates. Provides an evaluation at a macroscopic scale of the overall “OH-ness” of the system and facilitates an overall description of the system. Can be used pragmatically for preliminary assessment. Useful to identify key areas for improvement that can be evaluated into more details with a different tool.
Threats: This tool might be criticized because of	The tool is not well adapted to evaluation of complex surveillance systems that encompass multiple hazards and components, such as AMU and AMR, given that the surveillance of different AMR bacteria may differ in the same surveillance system. If results of evaluation or its application are not discussed with key people, its simplicity may lead to a superficial evaluation of certain aspects. Some indicators are not applicable to country or program context, e.g., added value of OH integration in the case a system was integrated from its beginning. Because data collection is expected to be short (e.g., no interviews), it is critical to have the right experts around the table to provide the required knowledge. Not suitable for end-users of the system. To ensure full comprehension of some indicators, previous clarification of their aim may be required, giving special attention to the terminology used, before conducting a meeting with relevant stakeholders. While the tool provides output figures describing the level of OH-ness, it does not allow to visualize the actual system (distribution of surveillance programs by sector and domain) or collaboration between actors/programs (e.g., via social network analysis). Adding this feature would be an asset.

## 4. Discussion

### 4.1. Overall perception of the tool

During the development of OH-EpiCap, several pilot applications on various surveillance activities were conducted. Due to the generic design of OH-EpiCap, it has been successfully applied to surveillance activities connected with food-borne hazards, such as *Salmonella*, *Campylobacter, Listeria*, and other zoonotic hazards such as *Chlamydia psittaci* (15). With this study, we illustrate its application to the evaluation of integrated surveillance systems for AMR.

According to the information collected in the nine case studies, OH-EpiCap can provide an overview of several crucial topics connected with AMR integrated surveillance, even though the tool was not specifically designed to evaluate these activities. The OH-EpiCap tool provides a summary assessment of the three dimensions targeted, which cover most of the elements that are important for assessing integrated surveillance systems, as described in the Integrated surveillance systems evaluation (ISSE) framework (5). The ISSE framework identified five levels of assessment for such surveillance systems, which include the integration of a OH approach, the production of integrated information and expertise, the generation of actionable knowledge, the influence on decision-making and the contribution to desirable outcomes. Evaluating these five levels in a comprehensive manner requires considerable time and resources, and OH-EpiCap constitutes a good first step toward evaluation of all of them.

Simplistic design and user friendliness, without requiring training of evaluators, are highly appreciated, not just by our assessors but also among users in general as shown in a survey recently undertaken among surveillance program practitioners and evaluators (16). To make the workshop more time efficient, it is recommended that at least one of the evaluators gets acquainted with the indicator questions and clarifies any possible doubts before organizing a session with the specialists involved in the evaluation and other relevant actors.

The outputs generated by OH-EpiCap may not lead directly to actions, however these can provide the basis for discussing further improvements with relevant stakeholders, as presented in a case study by (17). The MATRIX project also encompassed other activities that are complementary to the development of OH-EpiCap, such as a “Roadmap to develop national One Health Surveillance” which aims to function as a guideline for the development of OH Surveillance activities according to needs and resources in different countries (18).

An evaluation using OH-EpiCap can be conducted in a short period of time and with a small group of stakeholders, making it feasible to conduct an evaluation in situations with low resources. Moreover, evaluations can be done recurrently, when changes are implemented, benchmarking the system with itself over time. This can be made easily as OH-EpiCap contains benchmarking functionalities. These functionalities were not investigated in the present study, because of the different aims and purposes of the systems evaluated as noted in Table 2. For example, the Danish DANMAP program serves the purpose of integrated monitoring of AMU and AMR for both the animal and human sectors. In contrast, the Italian ClassyFarm encompasses mainly farm-level risk categorization components (e.g., biosecurity and animal welfare, besides AMR and AMU) whereas AMR surveillance in the human sector is conducted by different Italian institutions (19). Given the above-mentioned differences in the aims of the surveillance activities which we evaluated, indicator questions connected to real-time response capacity were considered not relevant in some surveillance activities.

AMR surveillance systems are complex and encompass multiple hazards, e.g., surveillance of clinical isolates in human health, bacterial isolates from animals at slaughter lines or in slurry, or AMR genes in sewage systems, each with their own particularities and logistics (5). So, when answering some of the questions representing an individual indicator in OH-EpiCap, interpretations need to be considered. This approach can justify the application of OH-EpiCap to several surveillance components, while focusing on one hazard at a time.

We applied the OH-EpiCap tool in nine different countries, by different native language users, providing important feedback to the developers regarding the phrasing of the indicator questions. We found that most of the indicator questions were considered simple and straight-forward. However, considering the expected worldwide application of the tool by users, who may have different use of the English language and, hence, familiarity with the terminology used, materials should be developed to unequivocally clarify the meaning of all indicators. With the publication of case studies evaluations and the scientific paper accompanying the tool (13), this should be accounted for. At the time of writing, the OH-EpiCap tool was still in a Beta Version, so the phrasing of indicators was not final.

### 4.2. Contribution of OH-EpiCap to the evaluation of integrated AMR surveillance systems

Except for Bangladesh, all country cases presented here were conducted in high-income countries. Hence, we have only limited experience regarding the applicability of the tool to low- and middle-income countries (LMICs). In LMICs, AMR surveillance is often hindered by deficient health system governance and restrictive financing of health data producing systems and laboratory capacities (20, 21). In addition, more efforts are needed to improve the capacity, quality standards, and integration of AMR surveillance in LMICs, which often have focus on human health. Due to its generic design, OH-EpiCap does not require that an integrated surveillance system is already established. However, at least primary surveillance activities need to be established and run. If this is not the case in a country, engagement in other tools such as the FAO Progressive Management Pathway for Antimicrobial Resistance (FAO-PMP-AMR), which aims to guide countries in the implementation of national action plans against AMR and early surveillance efforts (22), may be considered.

By highlighting components which may be improved in a hazard integrated surveillance system, OH-EpiCap can be considered as a valuable new addition to the current catalog of tools to evaluate integrated AMR surveillance systems (11). Moreover, OH-EpiCap can act as a simple gateway to conduct a more in-depth evaluation of certain surveillance system components as considered relevant. This may be done by using other pre-established tools designed to evaluate OH integration, such as the Evaluation of Collaboration for Surveillance (ECoSur) or The Network for Evaluation of One Health (NEOH).

The ECoSur tool has been developed to facilitate an in-depth analysis of the organization and functioning of collaboration taking place in a multisectoral surveillance system, aiming to evaluate the overall quality and relevance of such collaboration in meeting the objectives envisioned by stakeholders to produce the expected outputs of the program (23). From a user’s perspective, this tool gives a detailed evaluation of multisectoral collaboration in OH surveillance activities, however it requires a high level of abstraction to understand the indicator questions listed in the tool. Still, conducting a full ECoSur evaluation is rewarding regarding quality of output, but remains time and resource demanding (11).

The NEOH tool allows the evaluation of the coherence between operational and organizational aspects of OH activities, with the aim of identifying the added value of the integration across disciplines and sectors (24). From a user’s perspective, this tool is a comprehensive, multi-faceted fit for a transversal and detailed analysis of OH initiatives. However, conducting an evaluation using NEOH may be difficult and time consuming given that users should have specific training in systems thinking to make the most of it (11).

One of the ongoing activities in the CoEvalAMR network aims to simplify the application of the NEOH and ECoSur tools, using a modular approach. Given the complexity of evaluating integrated AMR surveillance systems, this could be of great value, targeting the evaluation to certain components which need to be prioritized.

Within the CoEvalAMR network, case studies have already been conducted from a user’s perspective on the application of the ECoSur (25) and NEOH tools (26–29). Other tools and frameworks that have been specifically designed to evaluate integrated AMR surveillance have also been evaluated: the FAO-PMP-AMR tool (30–33) as mentioned above (34); the FAO Assessment Tool for Laboratories and AMR Surveillance Systems (FAO-ATLASS) (35) developed to facilitate the assessment and definition of targets to improve national AMR surveillance systems in the food and agriculture sectors (36) and the ISSE framework (37, 38) developed to structure an assessment of the added value of integration in AMR surveillance systems (39). The interactive selection tool developed by the CoEvalAMR network can help users to select an appropriate tool for their needs (40).

## 5. Conclusion

The OH-EpiCap tool is a new addition to the portfolio of existing tools to evaluate integrated AMR surveillance systems. It provides a brief macro-overview of relevant OH topics, such as the perceived added value of establishing a OH team as a governance structure. This can serve as a basis to discuss possible adaptations of AMR surveillance activities, or targeting areas that may be further investigated using other established tools. It is free and easy to use, does not require training, and can be performed in less than a day provided that the group performing the evaluation has detailed knowledge on the surveillance system to be evaluated.

## Data availability statement

The original contributions presented in the study are included in the article/Supplementary material, further inquiries can be directed to the corresponding author.

## Author contributions

PM drafted the first version of the manuscript together with LA and LC. All other authors commented on the first version. PM took lead in revising the submitted manuscript together with LA and LC. All authors read and approved the final version of the revised manuscript and contributed with data from their national case study.

## Funding

This study was funded by the Canadian Institutes for Health Research through the Joint Programming Initiative on Antimicrobial Resistance (JPIAMR). The Portuguese case study was supported by funding from the European Union’s Horizon 2020 Research and Innovation programme under grant agreement No 773830: One Health European Joint Programme (MATRIX project).

## Conflict of interest

LC was involved in the development of the OH-EpiCap tool. LA works for an organization that gives advice to farmers and meat-producing companies.

The remaining authors declare that the research was conducted in the absence of any commercial or financial relationships that could be construed as a potential conflict of interest.

## Publisher’s note

All claims expressed in this article are solely those of the authors and do not necessarily represent those of their affiliated organizations, or those of the publisher, the editors and the reviewers. Any product that may be evaluated in this article, or claim that may be made by its manufacturer, is not guaranteed or endorsed by the publisher.
